# Furongian (Jiangshanian) occurrences of radiodonts in Poland and South China and the fossil record of the Hurdiidae

**DOI:** 10.7717/peerj.11800

**Published:** 2021-07-23

**Authors:** Xuejian Zhu, Rudy Lerosey-Aubril, Javier Ortega-Hernández

**Affiliations:** 1 Nanjing Institute of Geology and Palaeontology, Chinese Academy of Sciences, State Key Laboratory of Palaeobiology and Stratigraphy, Nanjing, China; 2 Harvard University, Department of Organismic and Evolutionary Biology and Museum of Comparative Zoology, Cambridge, MA, USA

**Keywords:** Furongian, Jiangshanian, Panarthropoda, Radiodonta, Hurdiidae, *Peytoia*, Burgess Shale-type preservation, Ediacara-type preservation

## Abstract

The Furongian period represents an important gap in the fossil record of most groups of non-biomineralizing organisms, owing to a scarcity of Konservat-Lagerstätten of that age. The most significant of these deposits, the Jiangshanian strata of the Sandu Formation near Guole Township (Guangxi, South China), have yielded a moderately abundant, but taxonomically diverse soft-bodied fossil assemblage, which provides rare insights into the evolution of marine life at that time. In this contribution, we report the first discovery of a radiodont fossil from the Guole Konservat-Lagerstätte. The specimen is an incomplete frontal appendage of a possibly new representative of the family Hurdiidae. It is tentatively interpreted as composed of seven podomeres, six of which bearing laminiform endites. The best preserved of these endites is especially long, and it bears short auxiliary spines that greatly vary in size. This is the second occurrence of hurdiids and more generally radiodonts in the Furongian, the first being the external mould of an oral cone from Jiangshanian strata of the Wiśniówka Sandstone Formation in Poland. Restudy of this Polish specimen confirms that it belongs to a hurdiid radiodont and best compares to *Peytoia*. The family Hurdiidae includes the oldest (basal Cambrian Epoch 2) and youngest (Early Ordovician, possibly Early Devonian) representatives of the Radiodonta and as such, has the longest stratigraphical range of the group. Yet, hurdiids only became prominent components of marine ecosystems during the middle Cambrian (Miaolingian), and their fossil record in younger strata remains limited.

## Introduction

The Cambrian period (541–485 Ma) is uncommonly rich in Konservat-Lagerstätten—deposits preserving fossils of both biomineralizing and non-biomineralizing organisms. These remarkable fossil localities provide unparalleled insights into the early evolution of animals, but their stratigraphic, geographical, and environmental distributions are uneven. The overwhelming majority of Cambrian Konservat-Lagerstätten, including the most studied ones (Caron & Rudkin, 2009; Zhao et al., 2011; Hu et al., 2013; Hou et al., 2017; Harper et al., 2019a), were deposited on the margins of the palaeocontinents Laurentia and South China, typically in quiet, relatively deep-water settings near the shelf break. Similarly, their stratigraphic distribution is heavily skewed towards the Cambrian Series 2 and the Miaolingian Series, whereas exceptional preservation is particularly scarce in Terreneuvian and Furongian strata (Muscente et al., 2017). The discoveries of Tremadocian exceptionally preserved fossils in the Floresta Formation in Argentina (Aris et al., 2016), the Dol-cyn-afon and Afon Gam Formations in Wales (Fortey & Rushton, 2003; Fortey & Rushton, 2009; Botting et al., 2015), and especially the Fezouata Shale in Morocco (Van Roy et al., 2010; Van Roy, Briggs & Gaines, 2015; Lefebvre et al., 2016) have highlighted the presence of a c. 13 Myr-long gap in the fossil record of macroscopic non-biomineralizing animals, which spans the Guzhangian and the whole Furongian Epoch. About 20 localities in the world have yielded macroscopic soft-bodied fossils of that age, but they remain unproductive and/or insufficiently collected (Lerosey-Aubril, 2017; Lerosey-Aubril et al., 2017a).

The most promising of these poorly studied Konservat-Lagerstätten is found in the Jiangshanian strata of the Sandu Formation in Guangxi, South China, and has yielded a taxonomically diverse fossil assemblage (c. 50 genera; Zhu et al., 2016). Although dominated by biomineralizing taxa (Han et al., 2000; Han & Chen, 2004; Han & Chen, 2008; Zhu, Hughes & Peng, 2007a; Zhu et al., 2007b; Zhu, Hughes & Peng, 2010; Zhu, Zamora & Lefebvre, 2014; Zhu et al., 2016; Zhan et al., 2010; Chen & Han, 2013; Zamora, Zhu & Lefebvre, 2013), the biota also comprises various ‘soft-bodied’ (i.e., weakly or non-biomineralizing) components: aglaspidid, mollisoniid, and ‘bivalved’ arthropods, the cnidarian *Sphenothallus*, one palaeoscolecid species, graptolites, algae, and taxa of indeterminate affinities (Lerosey-Aubril, Ortega-Hernández & Zhu, 2013; Lerosey-Aubril et al., 2017a; Zhu et al., 2016; Zhu, Lerosey-Aubril & Ortega-Hernández, 2019). The quality of preservation of these remarkable fossils greatly varies; some specimens exhibit exquisite anatomical details (e.g., fig. 3G in Zhu et al., 2016), whereas barely more than the outline is discernible in others (e.g., the appendage described herein). Also, the abundance of non-biomineralized fossils remains limited, which makes the Sandu Formation a typical Tier 3 Konservat-Lagerstätte *(sensu* Gaines, 2014). This classification is likely to change with time, as the thick (c. 2 km) Furongian succession exposed in the Guole area is explored and sampled. Meanwhile, each soft-bodied fossil discovered in the Sandu Formation adds to the considerably depauperate fossil record of non-biomineralizing organisms in the Furongian, and holds the promise of shedding new light onto the evolution of marine life in the Early Palaeozoic (Lerosey-Aubril, Zhu & Ortega-Hernández, 2017b).

In this contribution, we report the first discovery of a radiodont fossil—a frontal appendage—in the Sandu Formation. The Radiodonta is a taxonomically diverse order of extinct panarthropods that includes *Anomalocaris* and its relatives (Collins, 1996; Edgecombe, 2020). Traditionally regarded as free-swimming apex predators (Paterson et al., 2011), these organisms have proved remarkably diverse in size (millimetric to metric) and morphology, and are now reconstructed as having played various ecological roles in the early animal-dominated marine ecosystems (Daley & Budd, 2010; Daley & Edgecombe, 2014; Vinther et al., 2014; Van Roy, Daley & Briggs, 2015; Lerosey-Aubril & Pates, 2018; Liu et al., 2018; Moysiuk & Caron, 2019). Radiodonts are iconic of Cambrian Epoch 2–Miaolingian Burgess Shale-type biotas, each of which typically include several species (Pates et al., 2021a). Yet, their fossil record extends to the Ordovician, possibly the Devonian (Kühl, Briggs & Rust, 2009), although only one of the four families composing the group—the Hurdiidae—is known to occur in post-Miaolingian strata. Hurdiids are characterized by a well-developed tripartite cephalic carapace, and frontal appendages with three well-differentiated regions, the intermediate one bearing elongate, usually plate-like endites. The new frontal appendage from South China and an oral cone from the Wiśniówka Sandstone Formation in Poland constitute the only known occurrences of hurdiids and radiodonts in the Furongian Series. Their study provides us with an opportunity to critically review the hurdiid fossil record and to briefly discuss the possible impact of preservation bias on it.

### Geological setting

The hurdiid specimen was collected from calcareous mudstones of the Sandu Formation in the vicinity of Guole Township, Jingxi City, western Guangxi Zhuang Autonomous Region, South China. This lithostratigraphic unit is part of the Jiangnan Area, a transitional facies belt that extends along a SW-NE axis and separates the platform (NW) and basinal (SE) facies in the Furongian (Zhou, Zhen & Peng, 2008; Yao, Li & Li, 2015). The marl-dominated lithology of the Sandu Formation and its position within the Jiangnan Area suggest that it was deposited in the uppermost part of the continental slope (Zhu et al., 2016; Lerosey-Aubril, Zhu & Ortega-Hernández, 2017b). The Furongian deposits of the Guole area are essentially similar, both lithologically and palaeontologically, to those of the Sandu County (Guizhou Province) where the type section of the Sandu Formation is located. Accordingly, they are regarded as the same lithostratigraphic unit, rather than representing a distinct formation (‘Guole Formation’ of Han et al., 2000). At present, the thickness of the interval yielding exceptionally preserved fossils is unknown, but a Jiangshanian age for the Guole Biota can be inferred from trilobites (equivalent to the *Probinacunaspis nasalis*-*Peichiashania hunanensis* Zone of northwestern Hunan; Zhu, Hughes & Peng, 2010).

## Material and Methods

### Material

The radiodont material from the Sandu Formation described in this contribution consists of the part and counterpart of a single isolated frontal appendage, preserved compressed laterally and lacking its proximalmost and distalmost parts. This specimen was recovered at a new excavation site (locality 4) about 2 km NE of Guole Township (1 km E of locality 1 in Lerosey-Aubril, Zhu & Ortega-Hernández, 2017b). This fossil is deposited in the collections of the Nanjing Institute of Geology and Palaeontology, Chinese Academy of Sciences (NIGPAS 173694). The description of this new fossil is accompanied with a restudy of the only radiodont fossil hitherto known from the Furongian. This specimen (MWGUW ZI/66/0118) is the external mould of an oral cone from the Wiśniówka Sandstone Formation in Poland (Masiak & Żylińska, 1994). It is deposited in the collections of the Stanisław Józef Thugutt Museum of the Faculty of Geology, University of Warsaw, Poland.

### Illustration

Photographs of NIGPAS 173694 (dry) were taken using a Leica DFC420 digital camera mounted on a Leica MZ16 microscope. The image of the counterpart was mirrored using Photoshop CC to facilitate direct comparison between part and counterpart. Photographs of specimen MWGUW ZI/66/0118 (dry) were taken by M. Bieńkowska-Wasiluk; low-angle illumination from different directions was used to enhance the reliefs of its different parts. Interpretative drawings, one combining details of both parts of NIGPAS 173694 and the other of MWGUW ZI/66/0118, were produced based on pictures using Photoshop CC.

### Terminology

Many radiodont taxa have been described in the recent years, which leads to a constant reappraisal of the morphological variations exhibited by members of this order, and the most appropriate terms to describe it. As a consequence, there is no consensus yet regarding the best terminology to use in this group (e.g., Wu et al., 2021, table 1). The terminology employed in this contribution broadly follows the one used in the Hurdiidae diagnosis in Lerosey-Aubril & Pates (2018), and in Lerosey-Aubril et al. (2020). Importantly, the proximodistal tripartite organization of the frontal appendages in hurdiids is a diagnostic character of the family (Lerosey-Aubril & Pates, 2018). We believe that applying terms such as ‘shaft’/‘peduncle’ or ‘distal articulating region’, which are used to describe the bipartite frontal appendages of other radiodonts (e.g., Guo et al., 2019), to hurdiid frontal appendages may be counterproductive, for it obscures their fundamental difference in organization, overly complicates their descriptions (e.g., ‘proximal podomeres/endites of the distal articulated region’, instead of ‘intermediate podomeres/endites’), and carries the probably incorrect assumption that when comprised of several podomeres, the proximal part (‘shaft’/‘peduncle’) of the hurdiid appendage is not articulated (see Pates, Daley & Ortega-Hernández, 2017, fig. 1B for possible evidence of the contrary). Accordingly, we follow the terminology first introduced in the Hurdiidae diagnosis of Lerosey-Aubril & Pates (2018), and describe hurdiid frontal appendages as composed of: (1) a *proximal region* (or shaft/peduncle *sensu* Pates, Daley & Butterfield, 2019) typically composed of rarely more than one podomere (but see Lerosey-Aubril & Pates, 2018; Moysiuk & Caron, 2021), and usually associated with a single (absent in *Cambroraster*, possibly two in *Pahvantia*) distally-located endite that differs in size (narrower and usually shorter), shape, or orientation from intermediate endites; (2) an *intermediate region* of classically five or six podomeres (possibly up to eight podomeres in *Cordaticaris*; Sun, Zeng & Zhao, 2020b), which are associated with long (length of endite >height of corresponding podomere), typically laminiform endites; and (3) a *distal region* that includes up to six podomeres (e.g., *Stanleycaris*; Moysiuk & Caron, 2021) bearing much shorter, spiniform endites or no endites at all. In addition, when applied to the appendage: *proximal* and *distal* refer to the parts closest to or furthest from its insertion site on the body, respectively; *ventral* to the typically concave margin bearing the endites, and *dorsal* to the usually convex margin opposite to it. The height and length of the appendage or its podomeres refer to the dimensions along its dorso-ventral axis and proximo-distal axis, respectively (Briggs, 1979). When applied to an endite, *proximal* and *distal* are used to refer to the parts closest to or furthest from its insertion site on the appendage, respectively; *posterior* and *anterior* are then used to refer to the margin directed toward the proximal part or the distal part of the appendage, respectively (Briggs, 1979). Finally, a *laminiform* (‘plate-like’) endite is wide and flat, whereas a *spiniform* (‘spine-like’) endite is typically much narrower and presumably discoid or ovoid in section.

## Results

### A hurdiid frontal appendage from the Sandu Formation

#### Description

**Figure 1:**
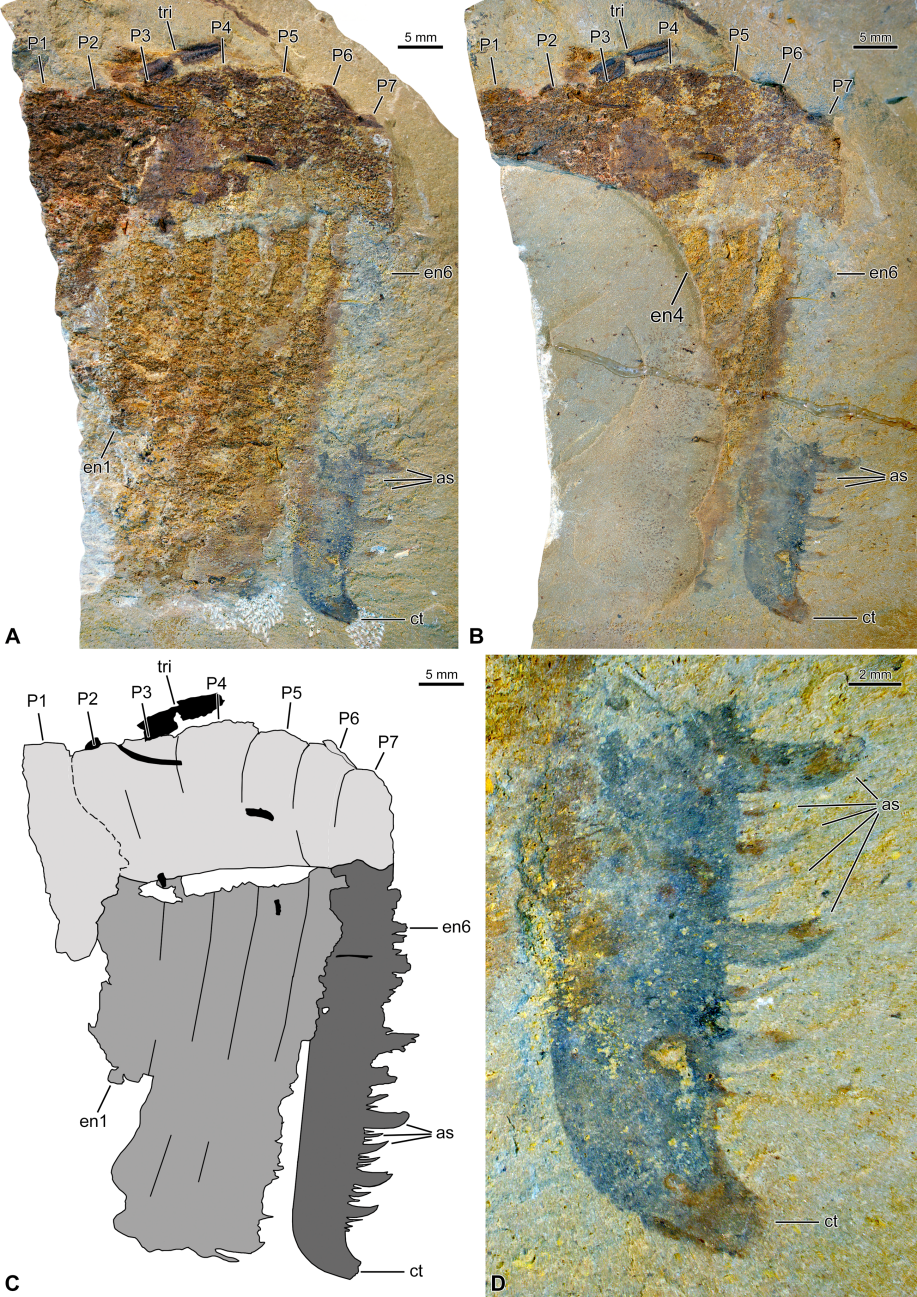
Hurdiid frontal appendage (NIGPAS 173694)** from the Cambrian (Jiangshanian) Sandu Formation, Guangxi, China. (A, B) General views of part (A) and counterpart (B; mirrored). (C) Interpretative drawing combining details of both parts (credit: Rudy Lerosey-Aubril). (D) Detailed view of distal part of endite 6 on counterpart, showing the size heterogeneity and truncated curved tips of the auxiliary spines. Abbreviations: *as*, auxiliary spine; *ct*, curved tip; *en*, endite; *P,* podomere; *tri*, trilobite fragment.

The incomplete frontal appendage measures 41 mm in length (along dorsal margin), and 16 mm and 10 mm in maximum (proximally) and minimum (distally) heights, respectively (endites excluded). The specimen is preserved compressed laterally and mostly composed of a rusty material, which probably represents iron oxides pseudomorphs after pyrite (Figs. 1A, 1B). This material has an especially coarse texture in the appendage proper, notably obscuring its segmentation. We tentatively reconstruct the appendage as composed of seven podomeres, based on incomplete podomere boundaries, indents of the dorsal margin, and the disposition and probable number of endites (Fig. 1C).

The proximalmost podomere (P1) seems devoid of hypertrophied laminiform endite and may therefore belong to the proximal region. All six remaining podomeres (P2–7) bear long, apparently unpaired, laminiform endites (one each; en1–6), and therefore belong to the intermediate region of the appendage. P2–4 are poorly delimited compared to P5 and P6, but there seems to be a general decrease in podomere length/height ratio distally; P7 is incomplete, but at least as long as P6.

En6 is the best preserved of the six visible endites and the only one to allow a relatively precise description of the morphology of intermediate endites in this taxon (Figs. 1A–1C). En6 is c. 47 mm long, and c. 75 mm wide all along, except for a slight narrowing near its distal tip. The latter region abruptly bends anteriorly to form a robust spine projecting at an angle of 110 ° relative to the long axis of the endite (Fig. 1D). Auxiliary spines only occur along the anterior margin of the endite. They greatly differ in size, but all are shorter than the endite width, form an angle of 90° with the endite margin, and curve distally, presumably towards the body midline in *in situ* position (Fig. 1D). Four sizes of auxiliary spines are tentatively recognized, the distribution of which seems to follow a repeated complex pattern along the proximo-distal axis of the endite: 41213121 (where 1 and 4 represent the smallest and the largest spines, respectively). This pattern is apparently repeated twice in the distal part of the appendage, but confirmation of its existence will await the discovery of additional specimens. Auxiliary spines on the more proximal part of en6 are visible on the part, but their insufficient preservation prevents a precise description of their distribution pattern. En1–5 are poorly preserved, but except for En1 that is incomplete distally, they look all similar in length, shape (essentially straight), and orientation (projecting at an angle of 100° from the appendage ventral margin, after compression).

#### Affinities

The new frontal appendage from Guole can be confidently assigned to the radiodont family Hurdiidae based on the presence of: (1) podomeres much taller than long; (2) six, partially overlapping, laminiform endites, among which at least five twice exceed in length the height of the podomeres bearing them; and (3) auxiliary spines on the anterior margins of the endites only (Lerosey-Aubril & Pates, 2018). Other radiodonts typically display endites that both alternate and decrease in size distally (anomalocaridids, amplectobeluids), or long spiniform endites bearing auxiliary spines on both their anterior and posterior margins (tamisiocaridids). The insufficient preservation of the new Chinese specimen prevents a confident assignment at a lower systematic rank, but it remains easily distinguishable from most representatives of the Hurdiidae (Table 1). The family includes the genera *Aegirocassis*, *Buccaspinea*, *Cambroraster*, *Cordaticaris*, *Hurdia*, *Pahvantia*, *Peytoia*, *Stanleycaris*, *Ursulinacaris*, and possibly *Schinderhannes* and *Zhenghecaris* (Pates et al., 2021a)*.* The endites of the frontal appendage from Guole are laminiform, unlike in *Ursulinacaris*, and narrowly-spaced, unlike in both *Stanleycaris* and *Ursulinacaris* (Pates, Daley & Ortega-Hernández, 2017; Pates, Daley & Ortega-Hernández, 2018a; Pates, Daley & Butterfield, 2019; Moysiuk & Caron, 2021). They bear auxiliary spines, not setae like *Aegirocassis* or *Pahvantia* (Van Roy, Daley & Briggs, 2015; Lerosey-Aubril & Pates, 2018), and these spines are apparently all shorter than the width of the endite they project from, which markedly differs from the conditions observed in *Buccaspinea* (Pates et al., 2021a), *Cambroraster* (Moysiuk & Caron, 2019), *Cordaticaris* (Sun, Zeng & Zhao, 2020a), *Schinderhannes* (Kühl, Briggs & Rust, 2009), and to a lesser extent *Hurdia* (Daley, Budd & Caron, 2013). If we are correct in inferring the presence of at least six laminiform endites in the Furongian frontal appendage (Figs. 1A, 1C), then again close relationships with *Aegirocassis* (Van Roy, Daley & Briggs, 2015), *Cambroraster* (Moysiuk & Caron, 2019), *Hurdia* (Daley, Budd & Caron, 2013), and *Stanleycaris* (Pates, Daley & Ortega-Hernández, 2018a) can be excluded, for all these taxa possess only five of such endites (Table 1). Overall, the frontal appendage from Guole best compares with frontal appendages assigned to *Peytoia*, a genus associated with an unusually diverse appendicular disparity, and in need of revision (Daley & Legg, 2015). The new Chinese frontal appendage is easily distinguished from forms of *Peytoia* with five or fewer laminiform endites, the distalmost of which or all lacking auxiliary spines (Daley, Budd & Caron, 2013, figs 13A–E; Daley & Legg, 2015). It is more reminiscent to *Peytoia* frontal appendages, sometimes referred to as ‘*Laggania*’ or ‘cf. *Peytoia*’, which possess six laminiform endites all bearing auxiliary spines (Briggs, 1979, pl. 80, figs. 1–4, 8, pl. 81, figs. 3, 8; Daley & Budd, 2010, text-figs. 7, 8; O’Brien, Caron & Gaines, 2014, fig. 7; O’Brien & Caron, 2016, fig. 2R; Moysiuk & Caron, 2021, fig. 6F, G). These auxiliary spines are short and greatly vary in size, but whether this size variation follows the same pattern than in the Guole frontal appendage is unclear. There has been little effort in detecting patterns of size variation of auxiliary spines along hurdiid endites, so whether this character would prove useful for the group systematics is uncertain. Accordingly, a more definitive assignment to the genus *Peytoia* of the hurdiid present in the Sandu Formation should await the discovery of additional specimens.

**Table 1:** Comparisons between the frontal appendages of hurdiids regarding key characters of the intermediate region.

**Characters**	**Intermediate endites**			**Auxiliary structures on distalmost intermediate endite**		**Key references**
	**Shape**	**Spacing**	**#**	**Type**	**Length**^a^	
**Guole appendage**	Laminiform	Tight	6 or more	Spines	Short	This study
***Aegirocassis***	Laminiform	Tight	**5**	**Setae**	**Long**	Van Roy, Daley & Briggs (2015)
***Buccaspinea***	Laminiform	Tight	6	Spines	**Long**	Pates et al. (2020)
***Cambroraster***	Laminiform	Tight	**5**	Spines	**Long**	Moysiuk & Caron (2019)
***Cordaticaris***	Laminiform	Tight	8 or more	Spines	**Long**	Sun, Zeng & Zhao (2020a)
***Hurdia***	Laminiform	Tight	**5**	Spines	**Medium**	Daley, Budd & Caron (2013); Daley et al. (2013)
***Pahvantia***	Laminiform	Tight	5 or more	**Setae**	**Long**	Lerosey-Aubril & Pates (2018)
***Peytoia*1**^b^	Laminiform	Tight	**5**	—	—	Daley, Budd & Caron (2013); Daley et al. (2013; fig. 13A-E)
***Peytoia*2**^b^	Laminiform	Tight	6	Spines	Short	Daley & Budd (2010; figs. 7, 8)
***Schinderhannes***	Laminiform	Tight	6	Spines	**Long**	Kühl, Briggs & Rust (2009)
***Stanleycaris***	Laminiform	**Wide**	**5**	Spines	Short	Moysiuk & Caron (2021)
***Ursulinacaris***	**Spiniform**	**Wide**	6	Spines	**—**	Pates, Daley & Butterfield (2019)

**Notes.**

Morphological differences between the frontal appendage from Guole and those of formally described hurdiids are highlighted in bold.

a Refers to the length of the longest auxiliary structure and whether it is shorter than endite width (*short*), equal to one to two endite width (*medium*), or longer than twice endite width (*long*).

b *Peytoia* 1 and *Peytoia* 2 refers to *Peytoia* frontal appendages with five laminiform endites (some lacking auxiliary spines) and six laminiform endites (all bearing auxiliary spines), respectively.

**Figure 2:**
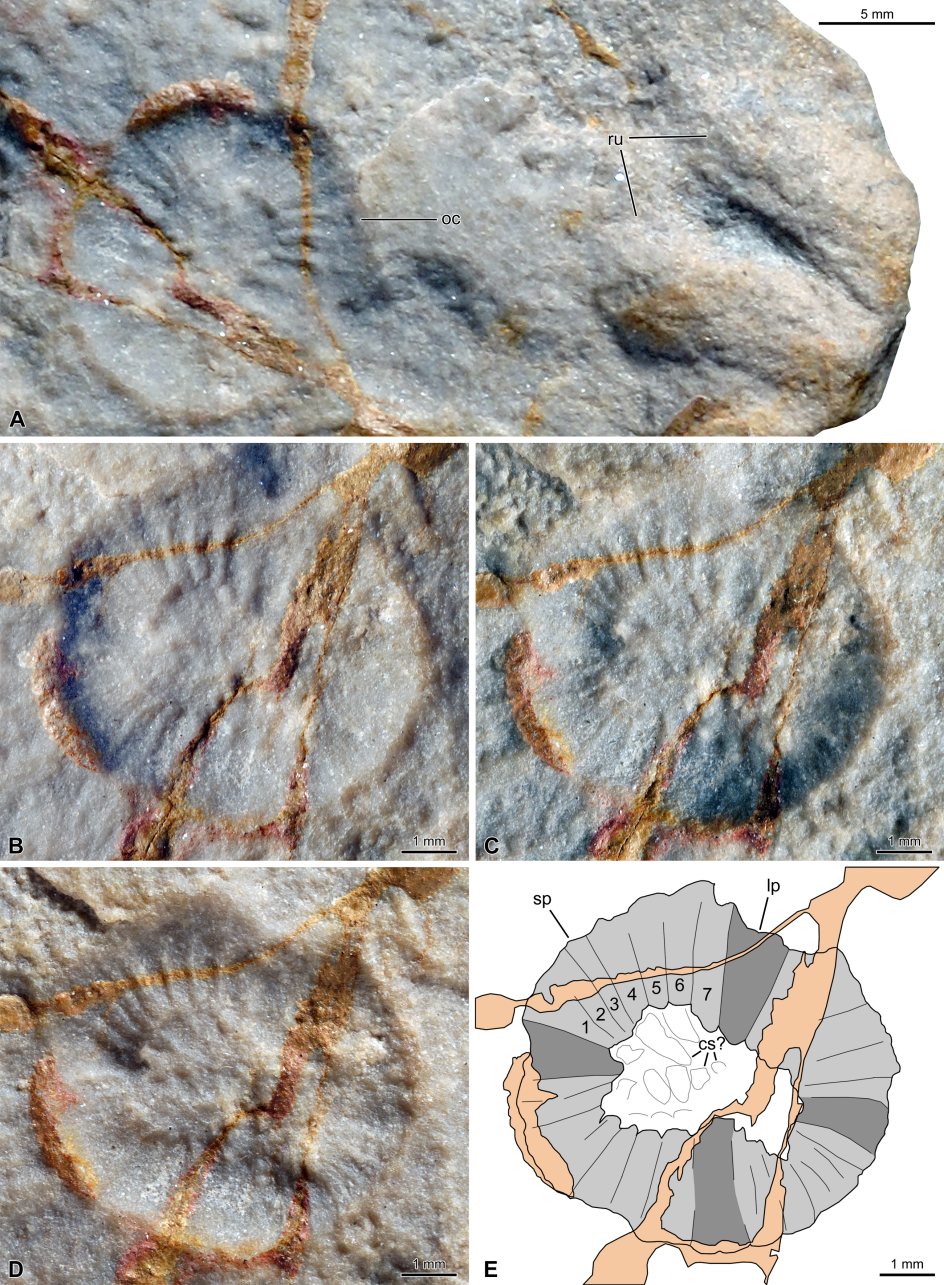
External mould of a *Peytoia* oral cone (MWGUW ZI/66/0118) from the Cambrian (Jiangshanian) Wiśniówka Sandstone Formation, Poland. (A) General view showing a *Rusophycus* trace fossil in positive relief next to the oral cone (specimen dry), which demonstrates that this surface is the sole of a bed. (B–C) Detailed views of the oral cone (specimen dry) with low-angle illumination from the left (B), bottom right (C), and bottom left (D); note that the oral cone is in negative relief (external mould). (E) Interpretative drawing of (B), (C), and (D) combined. Credits: Małgorzata Bieńkowska-Wasiluk (A–D) and Rudy Lerosey-Aubril (E). Abbreviations: *cs*, central structures; *lp*, large plate; *oc*, oral cone; *ru*, *Rusophycus* trace fossil; *sp*, small plate.

### The Jiangshanian oral cone from the Wiśniówka Sandstone Formation

#### Redescription

The redescription of this fossil, originally described by Masiak & Żylińska (1994), is based on new pictures provided to us by A. Żylińska and M. Bieńkowska-Wasiluk. MWGUW ZI/66/0118 is the external mould (negative relief) of an oral cone, which is subcircular in outline, particularly small (c. 7 mm in diameter), and composed of a circlet of 32 plates surrounding a subrectangular central opening (Figs. 2A–2E). Four, tetraradially arranged plates are notably larger than the others, but it is unclear whether size variation occurs between them, as depicted in hurdiids (Zeng et al., 2018a). The smaller plates are all similar in size and form sets of seven (*contra* Masiak & Żylińska, 1994), which separate the larger plates (most obvious in the upper left quarter of the circlet in Figs. 2B–2E). There is no evidence of folds along the outer margins of any of the plates, which all display a smooth surface. The absence of folds and nodes is interpreted as genuine, rather than resulting from preservation, for the grain size of the rock is noticeably smaller than the expected sizes of these structures, as inferred from published examples (e.g., Daley & Bergström, 2012; Zeng et al., 2018a; Sun, Zeng & Zhao, 2020a). However, the replication of the more central, originally deeper features is particularly limited compared to that of the more prominent parts of the oral cone, thus obscuring the morphology of the inner margins of the plates. Consequently, it is unclear how many marginal teeth may have projected from the inner margin of a given plate, as well as whether inner rows of plates occurred within the large central opening. The surface of the central area displays vaguely ovoid reliefs (Figs. 2B–2E), yet we doubt that they represent inner plates, for they are too large, not organized in rows, and somewhat comparable to reliefs visible at the surface of the slab some distance from the oral cone.

#### Affinities

This specimen was originally described as composed of 28 plates and tentatively assigned to the hurdiid genus *Peytoia* (‘*Peytoia*’ sp. of Masiak & Żylińska, 1994, and Żylińska, Szczepanik & Salwa, 2006), although Masiak & Żylińska (1994) noted that this number of plates differed from the condition known in *Peytoia nathorsti*. Our understanding of the morphological diversity of radiodont oral cones has considerably increased over the last 25 years, and families or even genera are now known to display unique combinations of circumoral features (Daley & Bergström, 2012; Zeng et al., 2018a; Liu et al., 2018; Sun, Zeng & Zhao, 2020a; Moysiuk & Caron, 2021). Our re-study shows that the Polish oral cone actually comprises 32 plates in total; it also considers some aspects of the morphology omitted in the original description that are necessary to re-assess the affinities of this fossil using modern standards.

**Figure 3:**
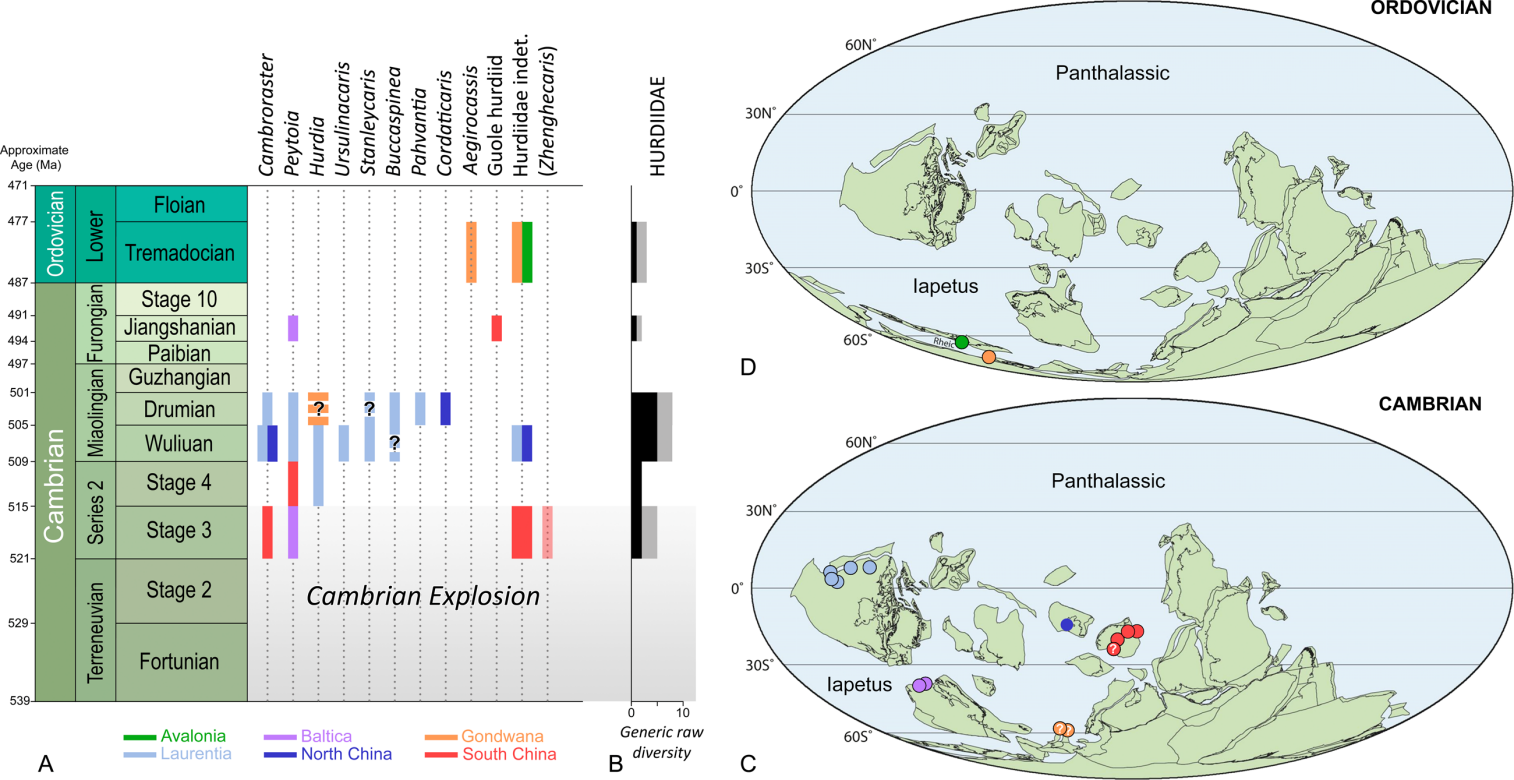
Stratigraphic ranges (A), taxonomic diversity (B), and palaeogeographical distributions in the Cambrian (C) and Ordovician (D) of hurdiid radiodonts. Note that *Schinderhannes*, a problematic taxon from the Lower Devonian of Germany, is not represented in the figure. *Zhenghecaris* is in parentheses to emphasize the fact that its hurdiid affinities are uncertain. The presence of *Cambroraster* in the Drumian of Laurentia is unequivocal, and based on half a dozen specimens from the Marjum Formation awaiting formal description (work in progress). Durations of geochronologic units and approximate ages of their boundaries are from Gradstein et al. (2020); ages were rounded up to the nearest million years. Background maps in (B, C) are from Torsvik & Cocks (2013).

The general shape of the fossil, the number of elements (32), and their organization as a circlet around a grossly rectangular opening support its interpretation as the oral cone of a radiodont. The presence of four large, perpendicularly arranged plates and four sets of seven, homogeneously sized smaller plates separating them exclude close affinities with Anomalocarididae (e.g., *Anomalocaris canadensis*, *A. saron*; Daley & Bergström, 2012; Zeng et al., 2018a). Similarly, the absence of outer folds and nodes on the plates speak against close relationships with amplectobeluids (e.g., ‘*Anomalocaris*’ *kunmingensis*, *Lyrarapax unguispinus*; Zeng et al., 2018a; Liu et al., 2018). Considering our present knowledge—the oral cones of tamisiocaridids and the peculiar radiodont *Caryosyntrips* are unknown—the Furongiame of the first ann specimen remains best assigned to the Hurdiidae and more specifically to the genus *Peytoia*, as originally tentatively proposed by Masiak & Żylińska (1994). Other representatives of this family, for which data on the oral cone are available, display 28 plates in total (*Stanleycaris; Moysiuk & Caron, 2021*), or nodes on the plates (*Cordaticaris, Stanleycaris*; Sun, Zeng & Zhao, 2020a; Moysiuk & Caron, 2021), or a subquadrate, rather than subrectangular central opening (e.g., *Cambroraster*, *Hurdia*; Zeng et al., 2018a; Sun, Zeng & Zhao, 2020a), features that do not characterize the Polish specimen. External nodes would be visible on an external mould, and a notable post-burial elongation of an originally square central opening would have resulted in the conspicuous elongation of some plates and the widening of others, which is not observed in the fossil. These two aspects of the morphology are thus considered as unaffected by preservation, and the genus *Peytoia* regarded as present in the Furongian of Baltica in our Fig. 3A.

## Discussion: Critical Reappraisal of the Fossil Record of Hurdiids

### Pre-Guzhangian evolution of hurdiids: a modest origin followed by a golden age

Most Cambrian exceptionally preserved faunas include radiodonts. Their remains—typically their more sclerotized body parts (i.e., frontal appendages, cephalic sclerites, oral cone)—are some of the first and most recognizable non-biomineralized fossils found in new remarkable localities (Liu et al., 2020). Most prolific Konservat-Lagerstätten (Tiers 1 and 2 of Gaines, 2014) contain between three to ten representatives of this clade, which usually exhibit marked morphological differences that reflect ecological partitioning (Daley & Budd, 2010; Pates et al., 2021a). The Hurdiidae has the longest temporal range (possibly up to 43 Myr according to Gradstein et al., 2020) of all radiodont families, which extends from the Cambrian Age 3 to the Early Ordovician (Tremadocian; Figs. 3A, 3B), maybe even reaching the Early Devonian (Emsian). Its oldest representative, *Peytoia infercambriensis* (Daley & Legg, 2015 and references therein), was recovered from Cambrian strata of Poland correlative with the lower *Schmidtiellus mickwitzi* Zone of other regions of Baltica (Moczydłowska, 2002), a trilobite biozone corresponding to the middle part of the Cambrian Stage 3 (Geyer, 2019; but see Moczydłowska & Yin, 2012 for an older estimate). Despite intriguing similarities with megacheiran frontal appendages (e.g., Aria et al., 2020), this fossil is usually regarded as the oldest occurrence of the order Radiodonta (Daley & Legg, 2015), a few million years older than the representatives of this group of panarthropods found in the Chengjiang and Sirius Passet Konservat-Lagerstätten.

Hurdiids are not known from the Greenlandic locality, but the recent description of a small carapace element assigned to the genus *Cambroraster* attests of their presence in the Chengjiang biota (Liu et al., 2020). *Zhenghecaris*, another Chengjiang taxon known from a couple of isolated sclerites, may represent an additional occurrence of the family in the Cambrian Stage 3 (Zeng et al., 2018b), along with a partial trunk from the same deposit (Hou, Bergström & Ahlberg, 1995, fig. 4) and possible carapace elements from the Shuijingtuo Formation (Cui & Huo, 1990; Daley, Budd & Caron, 2013). In the Cambrian Stage 4, hurdiids are known from a single frontal appendage of *Peytoia* from the Balang Formation of South China (Liu, 2013), and a disarticulated cephalic carapace of *Hurdia* from the Pioche Formation in the USA (Pates et al., 2021b); notably, they are conspicuously absent in two diverse biotas of that age, the Emu Bay Shale and Guanshan biotas (Daley et al., 2013; Jiao et al., 2021). In summary, the established fossil record of this radiodont family in the Cambrian Series 2 only consists of four fossils representing three genera (Figs. 3A–3C). This limited presence of hurdiids cannot be explained by a lack of Konservat-Lagerstätten, for the Cambrian Series 2 has yielded numerous exceptionally-preserved biotas (Muscente et al., 2017), some amongst the most diverse of the Cambrian (e.g., Chengjiang, Emu Bay Shale, Guanshan, Qingjiang, Sinsk, Sirius Passet). Besides, the other radiodont families are typically well-represented in these remarkable biotas (see table S1 in Zeng et al., 2018a), which points to the Hurdiidae truly being inconspicuous in the early Cambrian seas.

In contrast, the prominence of this radiodont family in Miaolingian strata is striking. No less than seven hurdiid genera are known in the Wuliuan, and a similar number are present in the Drumian (Figs. 3A, 3B). Remains of these organisms have been found in nine Konservat-Lagerstätten. The Burgess Shale biota shows the greatest generic diversity of hurdiids in the Wuliuan (four genera: *Cambroraster*, *Hurdia*, *Peytoia*, *Stanleycaris*; Daley & Budd, 2010 and references therein; Daley, Budd & Caron, 2013a; Pates, Daley & Ortega-Hernández, 2018a). Similar generic diversities, but incomparably lower abundances are observed in the Drumian Wheeler-House Range (*Buccaspinea, Pahvantia, Peytoia*, *Stanleycaris*) and Marjum (*Buccaspinea, Cambroraster*, *Pahvantia, Peytoia*) biotas of Utah (Pates, Daley & Ortega-Hernández, 2018a; Pates, Daley & Lieberman, 2018b; Lerosey-Aubril et al., 2020; Pates et al., 2021a). This greater taxonomic diversity of the group in the Miaolingian is all the more remarkable that it is almost exclusively documented by Laurentian biotas. Indeed, Miaolingian Konservat-Lagerstätten are particularly rare elsewhere in the world, which explains that the family is only known by one, possibly two occurrences in Gondwana (Czech Republic; Chlupáč & Kordule, 2002; Daley, Budd & Caron, 2013a; Mikuláš, Fatka & Szabad, 2012) and two occurrences in North China (Sun, Zeng & Zhao, 2020a; Sun, Zeng & Zhao, 2020b). It remains that with a total of eight genera, the Wuliuan-Drumian time interval apparently represents a *‘golden age’* for the Hurdiidae (Figs. 3A–3C).

### The Guzhangian–Cambrian Stage 10 interval: impact of preservation windows

The frontal appendage from Guole provides the first appendicular data for hurdiids for the time interval that spans the Guzhangian Age and the Furongian Epoch (Figs. 3A, 3B), and complements the particularly sparse fossil record of Radiodonta as a whole for that 13.6 Myr-long period (Gradstein et al., 2020). This record previously only included a handful of frontal appendages from the Guzhangian Weeks Formation (USA), assigned to two species of *Anomalocaris* (Lerosey-Aubril et al., 2014), and the oral cone from the Wiśniówka Sandstone, only mentioned in passing, if at all, after its first description by Masiak & Żylińska (1994).

The limited number of radiodont fossils in post-Drumian strata is not evidence of the group becoming rare, for all non-biomineralizing Cambrian taxa exhibit the same pattern at that time. This trend is traditionally interpreted as indicative of a substantial contraction of the Burgess Shale-type preservation window—the global development of marine conditions conducive to the preservation of carbonaceous remains during the Cambrian Epoch 2 and most of the Miaolingian (Gaines, 2014). In this regard, the preservation of the Wiśniówka oral cone is instructive, as it documents a unique mode of preservation for a radiodont fossil. This fossil is an external mould (negative relief) that is located on the sole of a sandstone slab, as attested by its association with a *Rusophycus* trace fossil in positive relief (Fig. 2A). To our knowledge, this is the only known example of Ediacara-type preservation (sensu Butterfield, 2003) in the fossil record of radiodonts, which is overwhelmingly composed of Burgess Shale-type fossils (for exceptions, see Gaines et al., 2012 and Kühl, Briggs & Rust, 2009).

The Ediacara-type preservation is the most common mode of fossilization of soft tissues in the late Ediacaran (MacGabhann, 2014), but it becomes exceedingly rare in the lower Cambrian (MacGabhann et al., 2019 and references therein). Guzhangian–Furongian strata apparently document a resurgence of this mode of preservation in the last 13.6 Myr of the Cambrian (Lerosey-Aubril, 2017), the oral cone from Poland (Baltica) being one among several examples around the world (Laurentia: Collette & Hagadorn, 2010; Siberia: Voropinov, 1957; Rozanov & Zhuravlev, 1992). These fossils are all found in relatively coarse-grained siliciclastic strata, which were deposited in proximal, shallow-water environments. Considering such lithofacies, in addition to the fine-grained distal deposits yielding Burgess Shale-type fossils (e.g., the Sandu Formation), might help filling the Furongian gap in the fossil record of non-biomineralizing organisms, including radiodonts.

### Post-Cambrian hurdiids: a hidden diversity?

Hurdiid fossils have been found in only two, or possibly three post-Cambrian Konservat-Lagerstätten (Figs. 3A, 3B, 3D). Only the Tremadocian part of the Fezouata Shale in Morocco has yielded abundant materials. These fossils have been overwhelmingly assigned to the giant suspension feeder *Aegirocassis benmoulai*, but illustrations of other hurdiid remains are scattered in the publications of Van Roy and colleagues, or their supplementary data (Van Roy et al., 2010; Gaines et al., 2012; Van Roy & Briggs, 2011; Van Roy, Daley & Briggs, 2015). Reviewing these lesser known specimens is a prerequisite to any assessment of hurdiid diversity in the Fezouata Shale. These fossils include a set of laminiform endites illustrated in Van Roy et al. (2010; fig. 1i), and a lateral carapace element, a dorsal one, and two isolated frontal appendages figured in Van Roy & Briggs (2011, fig. 1d–g, l and supplementary fig. S3c, d). The carapace elements best compare to *Hurdia*, but not the frontal appendages. One of the latter (Van Roy et al., 2010, fig. 1i) resembles a set of poorly preserved endites of *A. benmoulai*, although not identified as such in Van Roy, Daley & Briggs (2015). The other two published appendages (Van Roy & Briggs, 2011, fig. 1l and supplementary fig. S3c, d) likely belong to a new hurdiid, with characters reminiscent to amplectobeluids (e.g., long proximal region, massive dorsal spines). This atypical morphology may explain its basal position within the clade Hurdiidae in recent phylogenies (‘Fezouata hurdiid’ in Cong et al., 2014; Vinther et al., 2014; Van Roy, Daley & Briggs, 2015; Liu et al., 2018; Lerosey-Aubril & Pates, 2018). In summary, the Fezouata biota comprises two hurdiid genera, possibly three if the carapace elements and the second type of frontal appendages do not belong to the same taxon. Additionally, the Tremadocian fossil record of hurdiids includes a rare form from the Dol-cyn-Afon Formation in Wales, which is solely known from a tiny frontal appendage deemed unsuitable for the erection of a new taxon (Pates et al., 2020).

The youngest possible occurrence of the order and the family is represented by *Schinderhannes bartelsi* from the Lower Devonian (lower Emsian; De Baets et al., 2013) Hunsrück Lagerstätte (middle Kaub Formation) in Germany (Kühl, Briggs & Rust, 2009). Known from a single complete body, this taxon has been interpreted as combining characters typical of radiodonts (e.g., ‘frontal appendage’) and more crown-ward arthropods (e.g., trunk tergites and biramous appendages), along with a pair of large, enigmatic post-oral structures (for an alternative interpretation, see Ortega-Hernández, 2016). Originally recovered in an intermediate position between radiodonts and arthropods (Kühl, Briggs & Rust, 2009), *Schinderhannes* has been subsequently retrieved well-nested within the clade Radiodonta, as a hurdiid (Vinther et al., 2014; Van Roy, Daley & Briggs, 2015; Lerosey-Aubril & Pates, 2018; Liu et al., 2018; Moysiuk & Caron, 2019). If it were for the morphology of its pre-oral appendages alone, *Schinderhannes* would be confidently regarded as a hurdiid, but its trunk anatomy represents a significant departure from the radiodont body plan. Considering the lack of intermediate forms between *Schinderhannes* and Tremadocian radiodonts, and the 66 million years gap separating them (according to Gradstein et al., 2020), a hurdiid assignment of the Hunsrück animal should remain questionable.

To summarize, hurdiids, and more generally radiodonts, survived until (at least) the Tremadocian, but their presence in post-Cambrian strata is exceedingly rare. Investigating hurdiid diversity in the Fezouata Shale—the only known Ordovician Lagerstätte yielding abundant radiodont material—would allow to test whether this limited fossil record truly indicates the decline of hurdiids at that time or if it more likely results from a lack of remarkable marine fossil deposits.

## Conclusions

The Furongian period is associated with a decline of global marine biodiversity, which was recently called the ‘Furongian Biodiversity Gap’ (Muscente et al., 2018; Harper et al., 2019b). To some extent, this observed trend can be regarded as real (Harper et al., 2019b), and a consequence of the drastic fluctuations of environmental and climatic conditions characterizing this period (e.g., Saltzman et al., 2015). However, this decrease was exaggerated by a major disinterest for a time interval that postdates the Cambrian Explosion and predates the Great Ordovician Biodiversification (Lerosey-Aubril, 2017). This disinterest led to an insufficient exploration of Furongian rocks around the world and a significantly underestimated Furongian biodiversity, as recently demonstrated by Harper et al., (2019b). Until more attention is given to this pivotal period of the history of our planet, our understanding of the early diversification of marine animals and its dynamics (continuous *vs.* step-wise?) will remain severely impeded.

The radiodont fossils from the Sandu and Wiśniówka Sandstone formations illustrate how Konservat-Lagerstätten may contribute to the filling of the Furongian gap in the fossil record of non-biomineralizing organisms. These fossils represent the sole occurrences of the family Hurdiidae, and two of only four occurrences of the order Radiodonta in the strata representing the 14 last million years or so of the Cambrian. Despite originating in the basal Cambrian Epoch 2, hurdiids remains inconspicuous until Miaolingian times, when they become the most diverse radiodont components of exceptionally preserved biota. Occurrences of the family are rare again in post-Miaolingian strata, but this time mostly owing to a lack of Konservat-Lagerstätten associated with normal marine conditions.

## Additional Information and Declarations

### Data Availability

The following information was supplied regarding data availability: The specimen measurements and locations/numbers are available in the Description and Material and Methods sections. The fossils studied and illustrated in this contribution are deposited in the collections of the Nanjing Institute of Geology and Palaeontology, Chinese Academy of Sciences, Nanjing, China (NIGPAS 173694), and the Stanisław Józef Thugutt Museum of the Faculty of Geology, University of Warsaw, Warsaw, Poland (MWGUW ZI/66/0118). Measurements of these specimens are provided within their respective descriptions.
